# Gender-based violence among female youths in educational institutions of Sub-Saharan Africa: a systematic review and meta-analysis

**DOI:** 10.1186/s13643-019-0969-9

**Published:** 2019-02-25

**Authors:** Addisu Shunu Beyene, Catherine Chojenta, Hirbo Shore Roba, Alemu Sufa Melka, Deborah Loxton

**Affiliations:** 1 Research Centre for Generational Health and Ageing, Faculty of Health and Medicine, University of Newcastle, Newcastle, Australia; 2 School of Public Health, College of Health and Medical Sciences, Haramaya University, Harar, Ethiopia

**Keywords:** Gender-based violence, Youth, Educational institutions, Female, Sub-Saharan Africa, Systematic review, Meta-analyses

## Abstract

**Background:**

Gender-based violence is a public health issue. The prevalence of gender-based violence is high in Sub-Saharan Africa. Therefore, this study aims to produce an overall summary estimate on the prevalence of gender-based violence according to different types and its risk factors among female youths in educational institutions of Sub-Saharan Africa.

**Methods:**

Studies published in English between 2000 and 2017 were identified by searching electronic databases such as MEDLINE, CINAHL, EMBASE, PsychINFO, and other relevant data bases. Three reviewers independently extracted the data and assessed the quality of studies using the Loney guidelines. The pooled prevalence of gender-based violence and type of GBV was computed using STATA software version 14, and between studies heterogeneity was tested using Cochran’s *Q* test and *I*^2^ statistics. Meta-regression analyses were done to identify factors associated with GBV estimates.

**Results:**

A total of 1377 articles were produced from different databases, and a final 24 articles were included in the review. The overall prevalence of gender-based violence ranged from 42.3% in Nigeria to 67.7% in Ethiopia. The lifetime prevalence of sexual violence ranged from 4.3 to 76.4%, physical violence ranged from 7.4 to 66.1%, and emotional violence prevalence ranged from 26.1 to 50.8%. The overall pooled prevalence of lifetime GBV (*n* = 7 studies) was 52.83% [95% CI 39.54–65.90%, *I*^2^ = 99.1, *P* < 0.00]. The pooled estimate of sexual violence (*n* = 23), 26.22% [95% CI 19.48–33.57%, *I*^2^ = 99.39, *P* < 0.00], physical violence (*n* = 9), 18.86% [95% CI 10.96–28.3%, *I*^2^ = 98.98, *P* < 0.00], and emotional violence (*n* = 5), 27.06% [95% CI19.57–35.28%], *I*^2^ = 97.1, *P* < 0.00]. The review showed that gender-based violence was significantly associated with place of residence, witnessing parental violence, substance abuse, marital status, and educational status.

**Conclusions:**

The overall prevalence of overall gender-based violence, sexual, physical, and emotional violence was high in Sub-Saharan Africa. The lowest prevalence of GBV was observed in Nigeria, and it was highest in Ethiopia. However, the results should be interpreted with caution because of high between studies heterogeneity. Evidence from the review part revealed GBV was significantly associated with place of residence, witnessing parental violence, substance abuse, marital status, and educational status. The Sub-Saharan African countries should develop a comprehensive educational institution-based prevention strategy and effective interventions to mitigate gender-based violence and to specifically achieve the SDG_5_.

**Systematic review registration:**

PROSPERO CRD4201073260

**Electronic supplementary material:**

The online version of this article (10.1186/s13643-019-0969-9) contains supplementary material, which is available to authorized users.

## Introduction

Gender-based violence (GBV) is a major public health issue. It is projected that one in three women globally will face some form of abuse in childhood, adolescence, or adulthood [1–4]. GBV is now recognized as an important global public health problem because of its acute and chronic impacts on women’s health. GBV includes physical, sexual, and psychological abuse from intimate partners or non-partners [1–4]. The causes of gender-based violence are multi-dimensional, including social, economic, cultural, political, and religious factors [5].

According to the World Health Organization multi-country study on violence against women, the lifetime and current (past 12 months) prevalence of physical or sexual intimate partner violence ranged from 15 to 71% and 4 to 54%, respectively, and the prevalence of emotional violence ranged from 20 to 75% [6]. In another study conducted by the World Health Organization, it was estimated that the lifetime prevalence of intimate partner violence among female youths aged 15–19 was 29.4 and 31.6% for ages 20–24. The highest prevalence of intimate partner violence was reported in the African region, particularly in Sub-Saharan Africa (65.64%) [7]. Evidence from Sub-Saharan Africa (SSA) showed high rates of GBV in educational institutions. Results from the Global Based School Survey (GBSS) revealed that the magnitude of current physical and sexual violence in five African countries ranged from 27–50% and 9–33%, respectively [8, 9]. In research carried out in South Africa among adolescents aged 10–17 years, it was estimated that the lifetime prevalence (incident) of physical abuse was 56.3% (18.2%), emotional abuse 35.5% (12.1%), and sexual abuse 9% (5.3%) [10]. Researchers revealed the prevalence of attempted rape (18.7%), actual rape (23.4%), physically violent harassment (8.7%), verbal harassment (24.2%), and forced sexual initiation (11.2%) among female students in Wolaita Sodo University [11]. In other research, it was shown that the lifetime prevalence of rape was 11% among female secondary students in Arbamich [12].

In several studies, researchers showed that gender-based violence perpetration and victimization were associated with a combination of different factors. For example, age, rural residence, number of children, having witnessed family violence as a child, educational status, marital conflict, and partner and personal use of alcohol, tobacco products, and illicit drugs were the predictors of gender-based violence [9, 13–15].

GBV has been found to have detrimental effects on women, including injuries, sexual and reproductive health issues, mental health disorders, sexually transmitted infections (STIs), gynecological disorders, adverse pregnancy outcomes, an increased risk of non-communicable disease, and impacts on the health and wellbeing of their children [16, 17]. Another health effect of GBV is that it increases women’s risk of a number of other health problems, including chronic pain, physical disability, drug and alcohol abuse, and depression [18]. GBV also has a negative impact on a country’s human, social, and economic development and is an underlying obstacle to eliminating poverty and building peace [16, 17]. Students who had experienced gender-based violence were more likely to report low school achievement and an increased school dropout rate compared to non-abused youths [19, 20]. In a study conducted by WHO, it was found that schools and universities were highly vulnerable to GBV [21]. However, this problem is not well addressed in educational institutions [22].

As the above research has demonstrated, educational institutions are high risk spaces for GBV. This indicates that urgent intervention is needed to make educational institutions free of violence. To do this, systematically synthesized information is needed to design appropriate interventions and policies that target GBV in educational institutions in SSA.

Furthermore, over the last 20 years, a substantial amount of research on GBV has been conducted and published in SSA. However, the existing studies have not systematically identified and synthesized the prevalence of GBV among female youths in educational institutions [23]. Therefore, this study aims to produce an overall summary estimate on the prevalence of gender-based violence according to different types and its risk factors among female youths in educational institutions of Sub-Saharan Africa.

## Methods

### Protocols and registration

This systematic review has been registered in the International Prospective Registry of Systematic Review (PROSPERO registration number CRD4201073260, November 2, 2017). This systematic review followed the PRISMA guidelines [24].

### Eligibility criteria

The study focused on GBV (sexual, physical, and emotional/psychological violence) among young (10–24 years old) female students in educational institutions. Those studies which clearly reported the prevalence and risk factors for different types of GBV were included. The review included only studies conducted between 2000 and 2017 in SSA because most studies conducted in Africa focused on this area from the late 1990s in order to achieve the Millennium Development Goals, and no systematic review on GBV has been conducted in SSA among female youths in educational institutions. Published and unpublished papers were considered. The review considered studies involving female students in schools or universities or colleges using cross-sectional study design. The outcomes of interest were gender-based violence, sexual violence, physical violence, or psychological/emotional violence. Studies conducted in educational institutions using English were included in the review.

### Exclusion criteria

All studies published before 2000 and published in another language were excluded. Articles which did not have a full text or abstract were excluded. Community-based studies were excluded. Studies focusing on both males and females but which did not report separately were excluded. Studies that did not clearly report either prevalence or risk factors for gender-based violence among female youths in educational institutions were excluded.

### Information sources

The sources of the information were identified by searching electronic databases such as MEDLINE, EMBASE, PsychINFO, CINAHL, Google Scholar, and PubMed. The reference lists of identified articles were searched for additional studies. Furthermore, a hand search of key journals was conducted. Unpublished studies were searched in Google Scholar, universities’ online libraries, and government organization’s websites. We also made exhaustive efforts to contact authors to request the articles for which we did not have the full text or abstract or which reported the missing/incomplete data.

### Search strategy

The search strategy designed to access published and unpublished materials used the following search key terms and filters: “Gender based violence” OR “sexual violence” OR “physical violence” OR “psychological violence” OR “youth” OR “educational institutions” and “Sub-Saharan Africa” for each database. We consulted a librarian in designing the search strategy and searching the databases. The comprehensive database search was conducted on June 22–29, 2017. Further information regarding the search strategy of the selected databases is attached (see Additional file 1).

### Selection of the study

The study selection followed PRISMA flow diagram [24]. Endnote software was used to organize the papers. The relevance of the topic, objective, and methods of the study were checked. In the first stage, duplicates were removed. In the second stage, the title of the study was screened and those which did not meet the objective were excluded. In the third stage, abstracts of the studies were screened. Lastly, the contents of the remaining articles were assessed against the inclusion criteria.

### Data collection process

The Joanna Briggs Institute (JBI) data extraction form [25] was used by three reviewers (AB, HR, and AM) who independently extracted the data. Disagreement was resolved by discussion and consensus. If this was not possible, the matter was resolved by the fourth reviewer.

### Main data items

The extracted data included the authors, year of publication, country, sample size, sampling technique, type of educational institutions, outcomes, tools used to measure outcomes, and risk factors of specific studies.

### Quality of study

The quality of the studies and risk of bias were assessed by the checklist guidelines of Loney et al. [26]. The following criteria were used: (1) specified the target population, (2) used adequate sampling techniques (e.g., random), (3) adequate sample size (> 300 participants), (4) adequate response rate (≥ 80), (5) measurement with valid and tested instruments (Conflict Tact Scales 2 (CTS2) [27], WHO questionnaires [28]), (6) reported confidence intervals or standard errors, (7) reported attempt to reduce observer or other form of bias, and (8) study subject described in detail. The reviewers classified the tools into CTS2 and WHO questionnaires for assessing gender-based violence against females, and finally, “own tools” where unknown instruments were used (Additional file 2).

### Data synthesis

The individual studies were described concisely using a summary table. The summary table explained the characteristics of included studies and main findings. We used the random-effects model to pool the prevalence. The pooled lifetime prevalence of gender-based violence and type of GBV was computed using STATA version 14. Forest plot graphical representation and Cochran’s *Q* test and *I*^2^ were used to detect heterogeneity between the studies. Subgroup analyses were carried out to explore the potential sources of heterogeneity. Publication bias was checked using Eager’s weighted regression test [29]. Meta-regression analysis was carried out to identify parameters (publication year, quality score, and sample size) associated with GBV. The pooled estimate of lifetime prevalence was reported as an overall for GBV and by type of GBV (however, we decided not to use the result from meta-analysis except for the overall prevalence of GBV, sexual violence, physical violence, and emotional violence, due to high heterogeneity). The results were expressed qualitatively. We did not perform meta-analysis for risk factors due to the factors being varied among the studies. The results were described qualitatively. The findings of the review were presented in summarized tables, text, and a PRISMA flow diagram.

## Results

### Description of studies

**Fig. 1:**
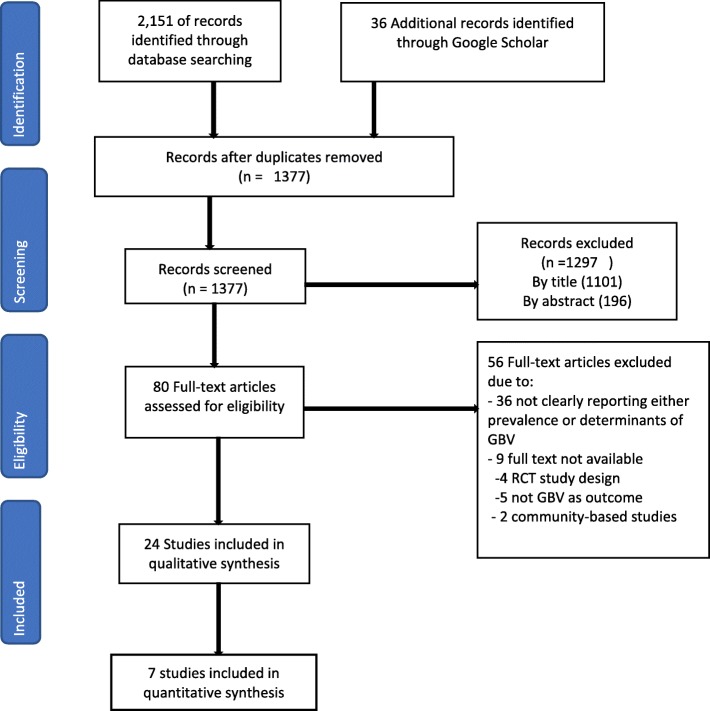
PRISMA flow chart for selection of publication

The search strategy produced 1377 articles from different databases and other sources after removing duplications (Fig. 1). Out of these, 1297 articles were excluded by reading the title and abstract due to their not being focused on gender-based violence. After further screening, 56 papers were excluded due to not clearly reporting either the prevalence or associated factors of gender-based violence. Eventually, 24 articles were reviewed for data analysis and interpretation. All articles included in this review were cross sectional. Two unpublished articles were included in this review [30, 31]. The sample size ranged from 140 to 25,840. The total number of participants was 41,334 (Additional file 3).

All studies specified the target population, used random sampling techniques, and attempted to reduce bias. Among 24 studies, 9 studies used WHO self-administered questionnaires, 3 papers used DHS questionnaires, 1 used the Childhood Experience of Care and Abuse Questionnaire (CECAQ), and 11 used self-administered questionnaires adapted from previous studies, and their own. The majority (*n* = 15) of studies were categorized as moderate quality (Additional file 2, Table 1). Seven studies investigated the overall prevalence of gender-based violence. The majority (*n* = 14) of studies exclusively focused on sexual violence. The settings for the majority of studies were Ethiopia, Nigeria, and Uganda (Additional file 3).

**Table 1:** Criteria used to assess the quality of studies

Quality item	No. of studies	Percentage (%)
Specification of the target population	24	100
Use of adequate sampling techniques	24	100
Adequate sample size (> 300 participants)	20	83
Adequate response rate (≥ 80)	24	100
Measurement with valid and tested instruments	9	38
Reported confidence intervals or standard errors	10	42
Reported attempt to reduce bias	24	100
Study subject described in detail	23	96

### Prevalence of GBV

**Fig. 2:**
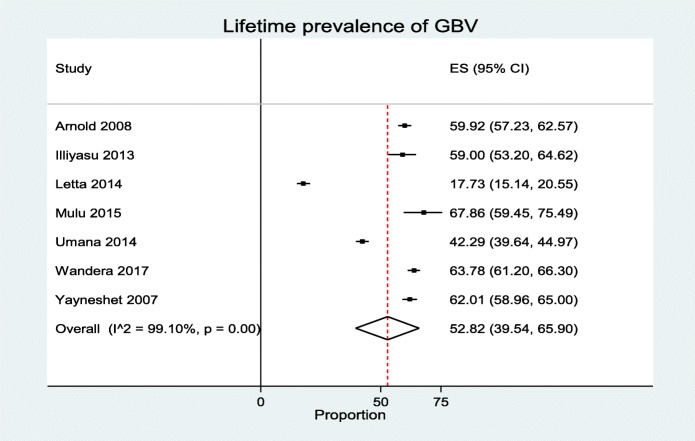
Pooled prevalence of gender-based violence (the diamond represents the overall combined pooled proportion and the square original studies proportion)

Seven studies with a total of 6347 participants, of whom these 3310 reported lifetime GBV, were included in the analysis. In these studies, researchers reported the overall prevalence of GBV ranging from 42.30 to 67.70% [31–37]. The pooled estimate prevalence of GBV was 52.83% [95%CI 39.54–65.90%]. The heterogeneity detected between the studies was (*I*^2^ = 99.10, *P* = 0.00), although Egger’s test showed non-significant for publication bias *P* = 0.55 (Fig. 2). Studies were further aggregated by study setting, publication year, quality score, and type of institutions. Subgroup analysis revealed the highest prevalence was in Uganda, 64.0% [95%CI 61.0–66.0%], while the lowest occurred in Nigeria, 45.0% [95%CI 43.0–48%]. The highest lifetime prevalence of GBV occurred in college students 61.0% [95%CI 59.0–63.0%], and the lowest occurred in university students 45.0% [95%CI 43.0–45.0%] (Table 2). Meta-regression analysis showed that the sample size, publication year, and quality score were not associated with GBV (Table 3).

**Table 2:** Subgroup analyses of the lifetime prevalence of GBV among female youths in educational institutions of SSA

Subgroup	Number of included studies	Prevalence (95% CI)
Study setting		
Ethiopia	4	51.0 [28.0, 74.0]
Uganda	1	64.0 [61.0, 66.0]
Nigeria	2	45.0 [43.0, 48.0]
Publication year		
< 2010	2	61.0 [59.0, 63.0]
> 2010	5	50.0 [31, 68.0]
Quality score		
Low	1	68.0 [59.0, 75.0]
Moderate	4	45.0 [25.0, 66.0]
High	2	61.0 [59.0, 63.0]
Type of institution		
Secondary	3	49.0 [16, 83]
College	2	61 [59, 63]
University	2	45 [43, 45]

*Note: GBV* gender-based violence, *SSA* Sub-Saharan Africa

**Table 3:** Meta-regression Analysis of covariates explaining sources of heterogeneity for the lifetime prevalence of GBV among female youths in educational institutions of SSA

Study characteristic	Number of studies included	Intercept	Coefficient	Standard Error	*P* value
Sample size	7	-.0000331	5598964	.0001558	0.840
Quality score	7	-.0173439	.6512461	.0816613	0.840
Publication year	7	-.0081695	16.97019	.020942	0.713

*Note: GBV* gender-based violence, *SSA* Sub-Saharan Africa

### Prevalence of sexual violence

**Fig. 3:**
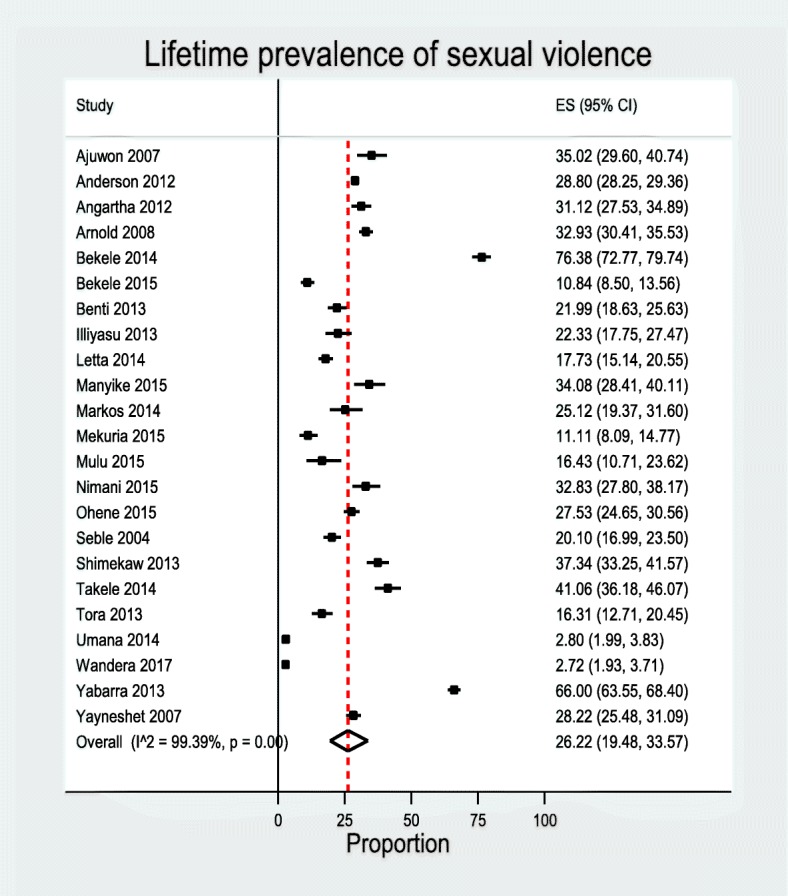
Pooled prevalence of sexual violence (the diamond represents the overall combined pooled proportion and the square represents the original studies proportion)

In 23 studies, researchers investigated the lifetime prevalence of sexual violence. A total of 40,409 participants with 11,511 cases of lifetime sexual violence were included in the analysis. The prevalence ranged from 4.30 to 76.40% [11, 12, 31–34, 36–51]. The pooled estimate prevalence of lifetime sexual violence was 26.22% [95%CI 19.48–33.57%]. The heterogeneity detected between the studies was (*I*^2^ = 99.39, *P* = 0.00), although Egger’s test showed non-significant publication bias *P* = 0.88 (Fig. 3). Subgroup analysis revealed the highest prevalence of lifetime sexual violence was in multiple countries, 29.0% [95%CI 28.0–29.0%] while the lowest occurred in Nigeria, 27.0% [95%CI 19.0–36.0]. The highest lifetime prevalence of sexual violence was among college students 30.0% [95%CI 25.0–36.0], and the lowest prevalence was similar among university students 25.0 [95%CI 10.0–45.0%] and secondary students 25.0% [95%CI 16.0–37.0%] (Table 4).

**Fig. 4:**
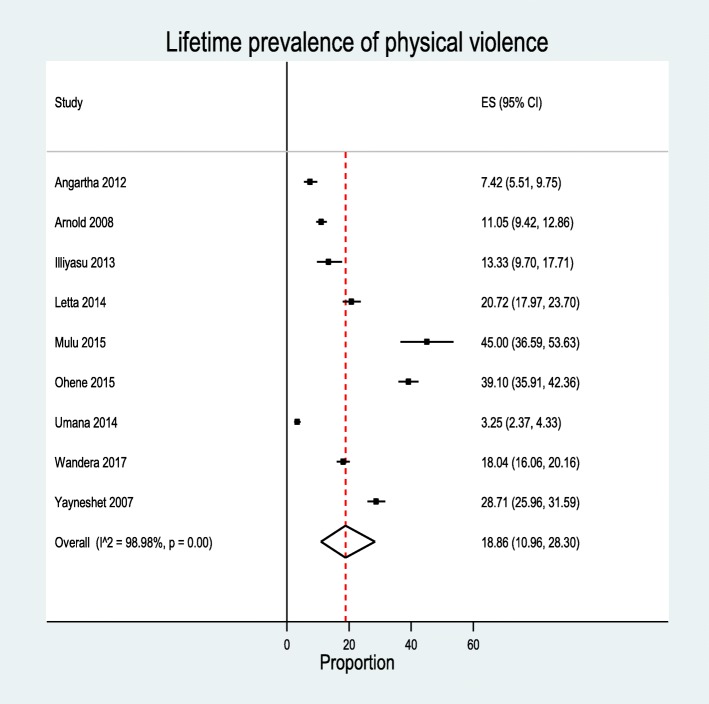
Pooled prevalence of Physical violence (the diamond represents the overall combined pooled proportion and the square represents the original studies proportion)

In four studies, researchers investigated the current prevalence of sexual violence. The prevalence ranged from 1.30% in Uganda to 57.7% in Ethiopia [31, 32, 37, 43]. In three studies, researchers investigated the prevalence of lifetime sexual coercion. The lifetime prevalence ranged from 35.0% in Nigeria to 76.4% in Ethiopia [39, 49, 52]. In two studies, researchers investigated the current prevalence of sexual coercion. The prevalence ranged from 25.4 to 43.7% in Ethiopia [49, 52]. In five studies, researchers found the prevalence of forced sex. The prevalence of completed rape ranged from 1.20 to 20.8% in Ethiopia, and the prevalence of attempted rape ranged from 4.2 to 27.5% in Ethiopia [11, 43, 47, 48, 51].

### Prevalence of harassment

In five studies, researchers investigated the prevalence of harassment. In two studies, researchers investigated the lifetime prevalence of any harassment, and prevalence ranged from 20.8 to 58% [48, 51]. In two studies, researchers investigated verbal sexual harassment and found the prevalence ranged from 18.7 to 90.4% [11, 53]. In two studies, researchers investigated the prevalence of physical harassment and found this ranged from 24.2 to 78.2% [11, 53]. In one study, researchers investigated the prevalence of non-verbal sexual harassment and found a prevalence of 90.4% [53]. In one study, researchers investigated the current rate of any harassment and found prevalence of 41.8% [48]. Surprisingly, all studies were from Ethiopia.

### Prevalence of physical violence

In nine studies, researchers investigated the prevalence of lifetime physical violence and found the prevalence ranged from 7.4 to 66.1% [31–38, 44]. The analysis included a total of 7888 participants and 1408 cases of lifetime physical violence. The overall prevalence of lifetime physical violence was 18.86% [95%CI 10.96–28.30%]. The heterogeneity detected between the studies was (*I*^2^ = 98.98, *P* = 0.00), although Egger’s test showed non-significant for publication bias *P* = 0.30 (Fig. 4). Subgroup analysis revealed the highest prevalence of lifetime physical violence (39.0%) was in Ghana [95%CI 36.0–42.0%] while the lowest (5.0%) occurred in Nigeria [95%CI 4–6.0%]. The highest lifetime prevalence of physical violence was among secondary students with a figure of 30.0% [95%CI 18.0–42.0%], and the lowest prevalence (7.0%) was among university students [95%CI 3.0–14.0%] (Table 4).

**Table 4:** Subgroup analyses of lifetime prevalence of sexual violence, physical violence, and emotional violence among female youths in educational institutions of SSA

Subgroup	Sexual violence		Physical violence		Emotional violence	
	*n* prevalence (95%CI)		*n* prevalence (95%CI)		*n* prevalence (95%CI)	
Study setting						
Ethiopia	14	27.0 [19.0, 36.0]	4	25.0 [14.0, 37.0]	1	25.0 [22.0, 28.0]
Uganda	3	29.0 [0, 77.0]	2	14.0 [13.0, 16.0]	2	22.0 [20.0, 24.0]
Nigeria	4	21 [4.0, 46.0]	2	5.0 [4.0, 6.0]	2	23.0 [21.0, 25.0]
Multiple	1	29.0 [28.0, 29.0]	–	–	–	–
Ghana	1	28.0 [25.0, 31.0]	1	39.0 [36.0, 42.0]	–	–
Publication year						
< 2010	3	29.0 [23.0, 35.0]	2	18.0 [16.0, 20.0]	–	–
> 2010	20	26.0 [18.0, 35.0]	7	19.0 [9.0, 31.0]	5	27.0 [20.0, 35.0]
Quality score						
Low	5	26.0 [19.0, 33.0]	1	45.0 [37.0, 54.0]	–	–
Moderate	13	25.0 [16.0, 35.0]	6	15.0 [6.0, 28.0]	5	27.0 [20.0, 35.0]
High	5	30.0 [0.12, 0.52]	2	18.0 [16.0, 20.0]	–	–
Type of institution						
Secondary	11	25.0 [16.0, 37.0]	4	30.0 [18.0, 42.0]	2	22.0 [20.0, 24.0]
College	4	30.0 [25.0, 36.0]	2	18.0 [16.0, 20.0]	–	–
University	8	25.0 [10.0, 45.0]	3	7.0 [3, 14]	3	31.0 [15.0, 48.0]

*Note: n* total number of studies included in the subgroup analyses, *CI* confidence interval

In four studies, researchers investigated the current prevalence of physical violence and found the prevalence ranged from 9.30% in Uganda to 54.80% in Ethiopia [31, 32, 35, 37].

### Prevalence of emotional violence

**Fig. 5:**
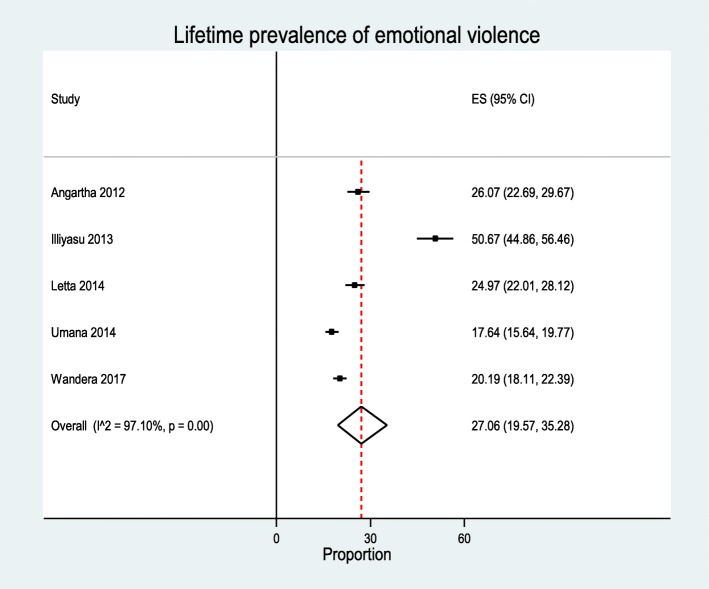
Pooled prevalence of emotional violence (the diamond represents the overall combined pooled proportion, and the square represents the original studies proportion)

In five studies, researchers investigated the prevalence of emotional violence and this ranged from 26.1 to 50.8% [33, 34, 36–38]. A total of 4486 study participants with 1038 cases of emotional violence were included in the analysis. The overall prevalence of emotional violence was 27.06% [95% CI 19.57–35.28%]. The heterogeneity detected between the studies was (*I*^2^ = 97.10, *P* = 0.00) although Egger’s test showed non-significant for publication bias *P* = 0.32 (Fig. 5). Subgroup analysis revealed the prevalence of lifetime emotional violence was the highest in Ethiopia, with 25.0% [95%CI 22.0–28.0%], while the lowest occurred in Uganda, with 22.0% [95%CI 20.0–24.0%]. The highest lifetime prevalence of emotional violence was among university students with a figure of 31.0% [95%CI 15.0–48.0%], and the lowest prevalence was among secondary students with 22.0% [95%CI [20.0–24.0%] (Table 4). In one study, researchers investigated the current prevalence of emotional violence and found a prevalence of 15.3% [37].

### Risk factors for GBV

In seven studies, researchers reported the overall prevalence of GBV, but in only two studies researchers showed the relationship between living arrangements, place of residence, and GBV [OR 1.60 for on campus [33], 1.43 for living far away from family [31]]. In three studies, a strong relationship between witnessing parental violence and GBV was shown [OR 2.40 [36], 2.20 [32], and 1.54 [31]].

Out of seven studies that investigated the overall prevalence of GBV, in four a relationship was found between alcohol consumption and GBV [OR 1.70 [32], 2.36 [36], 1.97 [31], and 0.38 [35]]. In one study, researchers reported those not drinking alcohol were less likely to report GBV [OR 0.38 [35]]. In one study, it was found that alcohol consumption and use of both alcohol and khat increased the risk of GBV [OR 1.70 and 1.76 [32]]. In another study, it was found that cigarette smoking and alcohol consumption were significantly associated with GBV [OR 2.46 and 2.36 [36]]. In one study, researchers reported that alcohol consumption, having drunken peers, having a boyfriend currently, being sexually active, and older increase the risk for GBV [OR 1.97, 2.63, 1.46, 1.44, and 1.71 [31], respectively].

In two studies, researchers showed evidence of a relationship between marital status and GBV [33, 36]**.** Those who were married was less likely to report gender-based violence [OR 0.53 [36]], and being single increased the risk of GBV [OR 1.51 [33]].

Out of seven studies which investigated the overall prevalence of GBV, in three studies, researchers found a relationship between educational status and GBV [31, 35, 36]. In two studies, it was found that postgraduate students, and those having a good academic performance, were less likely to report GBV [OR 0.64 [36] and 0.09 [35], respectively]. In one study, it was reported that poor school performance increased the odds of GBV [OR 2.09 [31]] (Addtional file 3).

### Risk factors for sexual violence

In two studies, researchers found that older age [OR 1.35 [46] and 1.79 [31]] was associated with sexual violence, but in another study, younger age [OR 1.71 [40]] was associated with sexual violence whereas another study found that women who were younger [OR 0.24 [49]] were less likely to report sexual violence. In one study, researchers found that being in grades 9–10 [OR 2.70 [45]] was associated with an increased risk of sexual violence compared with grade 12 and above. In several studies, parental education was implicated but not consistently. In one study, having a father who had completed primary or lower school [OR 3.06 [46] and 4.69 [12]] was associated with sexual violence as well, while the other study found that having a literate father [OR 0.17 [52]] was negatively associated with sexual violence. In yet another study, researchers found that having a mother whose education was less than grade four [OR 0.25 [41]] decreased the risk of sexual violence compared with illiterate mothers.

In two studies, researchers found that previous exposure to violence [OR 2.20 [38] and 1.84 [40]] was associated with sexual violence. In four studies, it was found that witnessing violence during childhood [OR 2.20 [32], 5.77 [41, 52], and 1.45 [31]] increased the risk of sexual violence, but in another study, it was found that witnessing parental violence [OR 0.49 [43]] decreased the odds of sexual violence.

In two studies, it was found that women living with both parents and a single parent [OR 0.56 for both parents, 0.47 for living with one parent [39], and 0.45 for both parents [48], respectively] were less likely to report sexual violence. In another two studies, researchers found that living with friends and living alone [OR3.32 for living with friends, 4.30 for living alone [12], and 4.90 for living with boyfriends [35]] increased the risk for sexual violence. In four studies, it was found that childhood rural residence [OR 1.60 [32, 33], 4.51 [47], and 1.48 [31]] was strongly associated with sexual violence.

In nine studies it was found that drinking alcohol [OR 1.80 [38], 1.31 [40], 1.80 [32], 4.20 [49], 2.31 [51], 1.53 [52], 5.66 [34], 7.30 [35], and 2.14 [31]] increased the odds of sexual violence. Similarly, in two studies researchers found that khat use [OR 3.11 [41] and 2.20 [32]] was associated with sexual violence.

In two studies, it was found that practicing sex with or without using a condom at recent sex [OR 2.41 with and 2.51 without [44], and 4.11 [46]] increased the risk of sexual violence. In three studies, it was found that being sexually active was significantly associated with sexual violence [OR 9.51 [51], 9.95 [48], and 1.82 [31]]. In another two studies, it was found that having multiple sexual partners [OR 4.32 [52] and 7.24 [51]] was associated with sexual violence.

In four studies, it was suggested that having delinquent peers [OR 4.19 [39], 2.70 [45], 4.78 [41], and 2.37 [48]] increased the risk for sexual violence. In another, researchers found that having peers that drank alcohol was significantly associated with sexual violence [OR 3.13 [47] and 1.98 [31]]. In one study, it was found that not having sexually active friends decreased the odds of sexual violence [OR 0.53 [44]], and in one study, it was also found that having a boyfriend/husband was negatively associated with sexual violence [OR 0.15 [43]].

In two studies, researchers found that having parents who were divorced or separated or widowed [OR 2.31 [48] and 3.04 [51]] was associated with sexual violence; conversely, in another study, it was found that having parents living together was associated with sexual violence [OR 6.53 [52]]. In one study, it was found that being single was associated with sexual violence [OR 1.51 [33]], and in another study, researchers found being married and divorced [OR 8.24 and 19.36 [34]] were contributing factors for sexual violence.

In three studies, it was found that receiving low pocket money [OR 6.95 [51]], earning an income from relatives [OR 4.37 [34]], and having an average family income ≤ 37.5 USD [OR 3.82 [12]] increased the risk for sexual violence. In one study, researchers found having social support from family decreased the odds of sexual violence [OR 0.89 [46]]. In another study, it was found students who lived near family and who had adequate money were less likely to report sexual violence [OR 0.13 [48]].

In one study, researchers found students who were monitored by their parents were less likely to report sexual violence [OR 0.82 [44]], and in one study, it was found students who did not have family supervision increased the odds of sexual violence [OR 1.92 [43]]. In two studies, researchers found that students not having discussed reproductive issues with parents were associated with sexual violence [OR 4.36 [47] and 2.93 [12]].

As demonstrated above, the factors associated with sexual violence were not consistent, with both older and younger ages, having literate or illiterate parents, witnessing and not witnessing familial violence, condom use and non-use, and separated/divorced and intact parental unions all had significant associations with sexual violence. Factors that were consistently associated with sexual violence included alcohol and khat use, peer sexual activity, and rurality (Addtional file 3).

### Risk factors for physical violence

In one study, it was found that physical violence was associated with high academic performance and high strength and difficulty questionnaire (SDQ) score [OR 1.36 and 1.05 [37]] while in one study, researchers found having poor academic performance was associated with physical violence [OR 2.51 [31]]. This shows students who have higher SDQ were more likely in turn to have a high risk of suffering from a mental health disorder. In one study, it was found that having an illiterate mother [OR 2.13 [32]] was associated with physical violence.

In three studies, researchers found witnessing parental violence as a child [OR 1.92 [37], 3.70 [38], and 1.87 [31]] was associated with an increased risk of physical violence. In one study, it was found that suicide attempts and the participant bullying others was associated with physical violence [OR 2.10 and 1.96 [44]].

In three studies, researchers found that living with stepmothers/fathers, living away from family, and being in a rural residence increased the risk of physical violence [OR 3.79 [34], 1.53 [31], and 4.85 [35], respectively]. In one study, it was found that physical violence was associated with living with biological parents and sharing a sleeping area with one adult [OR 1.17 and 1.23 [37]]. In another study, researchers found that girls whose parents respected their privacy were less likely to report physical violence [OR 0.83 [44]].

In three studies, it was found that students using alcohol [OR 1.90 [38], 2.25 [44], and 3.80 [34]] had increased odds of physical violence. In one study, researchers found having boy/girl friends who drank alcohol was associated with physical violence [OR 1.98 [31]].

As demonstrated above, the factors associated with physical violence were not consistent. Both high and poor academic performance and living with single parents and both parents all have significant associations with physical violence. Factors that were consistently associated with physical violence included witnessing parental violence, alcohol use, having boys/girl’s friends who drank alcohol, and rurality (Additional file 3)

### Risk factors for emotional violence

In two studies, researchers found that alcohol users [OR 2.20 [38] and 2.99 [34]] were more likely to report emotional violence. In one study, it was found that witnessing parents’ violence as a child and having an attitude supportive of violence against children from school staff was associated with emotional violence [OR 1.29 and 1.04 [37]].

In one study, researchers found that living with biological parents, walking alone or with someone to school, and having a high SDQ score was associated with emotional violence [OR 1.22, 1.68, 1.83, and 1.05 [37], respectively]. In one study, it was found emotional violence was associated with having ever worked for payment [OR 1.36 [37]]. In another study, researchers found that having a mother whose occupation was a farmer and owning a private business increases the risk of emotional violence [OR 3.68 and 1.76 [34]].

As shown above, the factors that were consistently associated with emotional violence included witnessing parental violence, alcohol use, living with both parents, walking alone or with someone, and occupation of mothers (Additional file 3).

## Discussion

In Africa, studies on the prevalence and risk factors for physical and emotional violence are limited but sexual violence has been exhaustively investigated. Studies in Africa are highly heterogeneous due to the definitions used, sample size, tools used, and data collection methods, and more importantly, the culture, norms, and values of the studies varied. Therefore, it is difficult to generalize the results**.**

Despite these variations, this review found that the overall prevalence of gender-based violence in Sub-Saharan Africa was high, ranging from 42.3 to 67.7%. This finding was relatively similar with the finding from an analysis of WHO data in 2014 among adolescents and young women that ranged from 19.0 to 66.0%) [54]. This may be due to the sample size and composition of participants. The majority of studies used their own self-administered questions and not standardized cross samples. These questions may not have been validated for use with the populations that were sampled. This may lead to overestimates or underestimates of the prevalence of GBV.

The lifetime prevalence of sexual violence ranged from 4.30 to 76.40%. This wide range may be due to definitions and tools used to measure sexual violence across studies, as well as the differing samples. The majority of studies measured sexual violence using a single item. This may have led to underreporting or overreporting of the prevalence of sexual violence. This finding is higher than a meta-analysis conducted in 2011 that examined the global perspective of child sexual abuse (18.0%) [55], a worldwide systematic review and meta-analysis conducted on sexual violence (15.0%) [56], and in research that used a community sample (19.4%) [57]. The difference may be due to sample size, composition, and definitions and tools used to measure sexual violence. The sampled population varied from high school students to university students; therefore, the prevalence of sexual violence was varied.

The lifetime prevalence of physical violence ranged from 7.4 to 66.1%. However, there was high variation among the studies. These variations may have been due to culture, norms, socioeconomic factors, and definitions and tools used to measure physical violence. These variations may lead to underreporting or overreporting of the prevalence of physical violence. The other possible explanation may be women in developing countries justify the fact that men beating their partner is acceptable; males dominating females is prevalent in Africa [58]. Additionally, the majority of these systematic reviews used their own self-reporting tools, and the majority of studies used various definitions of physical violence and measured prevalence using single items as well as differing samples.

The lifetime prevalence of emotional violence ranged from 26.1 to 50.8%. However, there was a high degree of variation between studies. The differences between studies might be because of different culture, societal factors, and economic status. Studies used various definitions of emotional violence. Only five studies reported emotional violence. This indicates a lack of studies on this topic.

This systematic review also identified different factors associated with GBV. Living arrangements were associated with GBV [31, 33]. This is consistent with studies conducted in other parts of the world [9, 13, 59, 60]. This might be due to rural residents perhaps not having access to health information and infrastructure. This review found that witnessing parental violence was a risk factor for GBV [31, 32, 36]. This is similar with the studies carried out around the globe [9, 13, 14, 54, 61].

This review demonstrated that substance use was a contributing factor for GBV [31, 32, 35, 36]. This is concomitant with the previous studies [8, 9, 13, 14, 54]. This may be due to alcohol influencing decision making, which may lead to GBV. Marital status was also found to be a risk factor of GBV [34, 36]. This is in line with previous studies around the globe [9, 13, 14]. This review also found that educational status was associated with GBV (three studies). This corroborates with previous studies in other parts of the world [9, 13, 59, 60]. This might be due to educated women having more of an awareness of GBV than illiterate women.

This review showed a strong relationship between witnessing parental violence during childhood, and sexual violence [31, 32, 41, 50]. This finding corresponds with a study from the USA [62, 63]. Being a rural resident was found to be a risk factor for sexual violence [31–33, 47]. Alcohol consumption was the most common factor associated with sexual violence [31, 32, 34, 35, 38, 40, 49–51]. This is supported by the evidence from high-income countries [64, 65]. Being sexually active was associated with sexual violence [31, 48, 51]. This association was also found in studies in high income countries [63]. This review also found having peers was a contributing factor for sexual violence [39, 41, 45, 48]. This corresponds with a study from high-income countries [63]. One possible explanation may be the persons may not be interested to reject by their peers.

Witnessing parental violence during childhood was found to be associated with physical violence [31, 35, 37]. This is consistent with a study from a high-income country [66]. Alcohol drinking was found to be associated with physical violence [34, 38, 44]. This corroborates with previous studies across the world [64].

This review also found factors associated with emotional violence. Alcohol consumption was found to be a risk factor for emotional violence [34, 38]. This is consistent with studies around the globe [64, 65, 67]. Witnessing parental violence was also found to be a contributing factor for emotional violence [37]. This was also evidenced by meta-analytic review [68].

Furthermore, this systematic review showed that witnessing violence inside the home is associated with GBV in the educational setting. Those students who have witnessed violence at home also had increased risks of experiencing sexual, physical violence, and emotional violence in the educational setting. This might be due to continuous disagreement between their parents/caregivers. This has intergenerational consequences. The other possible explanation may be students who have witnessed violence at home have poor parental supervision and less parental support.

### Implications of the study

Even though there was a high degree of heterogeneity between studies, GBV is still a significant public health problem. This reveals that GBV is common among youths in educational institutions of SSA. This review has implications for the design of intervention, policy, and programming in SSA. It also suggests that a youth violence prevention policy, intervention strategies, and service provisions are needed in SSA. GBV has a detrimental effect on youths, but especially women and girls, and is an obstacle to achieving the Sustainable Development Goals (SDGs). In order to achieve Sustainable Development Goal 5 (gender equality), eliminating GBV in educational institutions and the community through educating the community/parents and students about the prevalence, causes, and consequences of problem is crucial. This review will also help government policy makers, non-government organizations, and other stakeholders to alleviate the burdens of gender-based violence. All Sub-Saharan African countries should develop a community and school-based intervention program to address gender-based violence against youths at educational institutions and community settings.

### Weakness and strengths of study

There was high heterogeneity between studies. This systematic review used institutional-based cross-sectional survey, and so causality cannot be established. The review did not include youths who do not attend school. Additionally, the majority of studies did not use the international standardized questionnaire. The majority of studies were of moderate quality. They may be subject to recall and social desirability bias.

## Conclusion and Recommendation

This systematic review found that the prevalence of overall gender-based violence, sexual, physical, and emotional violence was high. However, the results should be interpreted with caution because of high between-study heterogeneity. This review also found that living arrangements, educational status, marital status, witnessing parental violence, substance use, sexual risk factors, and peer pressure was strongly associated with gender-based violence. The results highlight the need for government policy makers, non-governmental organizations, program designers and other stakeholders to develop effective intervention and prevention strategies, and programs to reduce gender-based violence in educational institutions. A comprehensive educational institution-based prevention strategy and effective interventions should be developed to mitigate gender-based violence. We also identified a paucity of studies examining emotional violence in the educational setting in Sub-Saharan Africa. The authors recommend that further studies should be carried out by using a longitudinal study on gender-based violence among in-school and out-school youths in order to establish causality. The future study should focus on ploy-victimization of youths in SSA. Additionally, future research should focus on the culture of the community to identify the risk and protected factors of gender-based violence among youths in SSA. Moreover, due to the high heterogeneity of the included studies, future GBV studies undertaken across SSA should utilize a standardized methodology which could allow for comparisons to be made over time.

## Additional files

Additional file 1: Searching strategy. (DOCX 20 kb)

Additional file 2: Quality assessment. (DOCX 18 kb)

Additional file 3: Description of studies included in the review. (DOCX 34 kb)

## Abbreviations

AOR: Adjusted odd ratio
CI: Confidence intervals
GBV: Gender-based violence
OR: Odds ratio
PRISMA: Preferred Reporting Items for Systematic Reviews and Meta-Analyses Protocols
SDGs: Sustainable Development Goals
SSA: Sub-Saharan Africa
WHO: World Health Organization
