# Supplementary material for: Gender-based violence among female youths in educational institutions of Sub-Saharan Africa: a systematic review and meta-analysis
Source: Syst Rev. 2019 Feb 25;8:59. doi: 10.1186/s13643-019-0969-9 (PMC6388495; doi:10.1186/s13643-019-0969-9)
Supplement: Supplementary file 1 — Searching strategy. (DOCX 20 kb) [file 13643_2019_969_MOESM1_ESM.docx]

Additional file 1: Searching strategy

Searching strategy used to identify relevant studies for review.

“Gender based violence”, “sexual violence”, “physical violence”, “emotional violence”, “psychological violence”, “assault”, ‘abuse”, “bullying”, “intimidation”, “rape”, “violence”, “battering”, “stalking”, “injury”, “beating”, “incest”, “harassment”, “youth”, “adolescents”, ”teenager“, “young people”, ”young adult”, ”educational institutions”, “college”, “university”, “high school”, “secondary school”, “preparatory school” and “Sub-Saharan Africa”.

OR

| Terms and filters used | Data bases | Numbers of final obtained |
| --- | --- | --- |
| “Gender based violence OR Sexual violence OR Physical violence OR Psychological violence OR Educational Institution OR Female AND Sub-Saharan Africa” commenced from 22-29 June, 2017 | Medline, Psych Info, Embase | 1917 articles |
| “Gender based violence OR Sexual violence OR Physical violence OR Psychological violence OR Educational Institution OR Female AND Sub-Saharan Africa” commenced from 22-29 June, 2017 | Pubmed | 221 articles |
| “Gender based violence OR Sexual violence OR Physical violence OR Psychological violence OR Educational Institution OR Female AND Sub-Saharan Africa” commenced from 22-29 June, 2017 | CINAHL | 13 articles |
| “Gender based violence OR Sexual violence OR Physical violence OR Psychological violence OR Educational Institution OR Female AND Sub-Saharan Africa” commenced from 22-29 June, 2017 | Google scholar | 36 articles |
| **Final included articles in review** | | **24 articles** |
