# Supplementary material for: Gender-based violence among female youths in educational institutions of Sub-Saharan Africa: a systematic review and meta-analysis
Source: Syst Rev. 2019 Feb 25;8:59. doi: 10.1186/s13643-019-0969-9 (PMC6388495; doi:10.1186/s13643-019-0969-9)
Supplement: Supplementary file 2 — Quality assessment. (DOCX 18 kb) [file 13643_2019_969_MOESM2_ESM.docx]

Additional file 2: Quality assessment

Table 6 Quality assessment studies

| Authors | Quality item | | | | | | | | |
| --- | --- | --- | --- | --- | --- | --- | --- | --- | --- |
|  | Specification of the target population | Use of adequate sampling techniques | Adequate sample size (>300 participants); | Adequate response rate (>=80) | Measurement with valid and tested instruments | Reported confidence intervals or standard errors | Reported attempt to reduce bias | Study subject describe in detail | Total score (8) |
| Agardh  et.al (2012) | Y | Y | Y | Y | N | N | Y | Y | 6 |
| Ajuwon A. et.al (2006) | Y | Y | N | Y | N | N | Y | Y | 5 |
| Anderson et.al. (2012) | Y | Y | Y | Y | N | N | Y | Y | 6 |
| Markos et.al  (2014) | Y | Y | N | Y | N | N | Y | N | 4 |
| Ohene et.al  (2015) | Y | Y | Y | Y | Y | N | Y | Y | 7 |
| Wandera et.al (2017) | Y | Y | Y | Y | Y | N | Y | Y | 7 |
| Yabarra et.al  (2013) | Y | Y | Y | Y | N | N | Y | Y | 6 |
| Arnold et al  (2008). | Y | Y | Y | Y | Y | Y | Y | Y | 8 |
| Takle Abulie and Tesfaye setegn  (2014) | Y | Y | Y | Y | N | N | Y | Y | 6 |
| Bekele et.al  (2015) | Y | Y | Y | Y | Y | N | Y | Y | 7 |
| Bekele et.al  (2014) | Y | Y | Y | Y | Y | Y | Y | Y | 8 |
| Benti Tsegaye and Teferi Elias (2015) | Y | Y | Y | Y | N | Y | Y | Y | 7 |
| Iliyasu et.al  (2011) | Y | Y | Y | Y | N | Y | Y | Y | 7 |
| Mamaru et.al  (2015) | Y | Y | Y | Y | Y | N | Y | Y | 7 |
| Letta et.al  (2014) | Y | Y | Y | Y | N | Y | Y | Y | 7 |
| Mullu et.al  (2015) | Y | Y | N | Y | N | N | Y | Y | 5 |
| Mekuria et.al  (2015) | Y | Y | Y | Y | Y | Y | Y | Y | 8 |
| Shimekaw et.al  (2013) | Y | Y | Y | Y | N | Y | Y | Y | 7 |
| Umana et.al  (2014) | Y | Y | Y | Y | Y | N | Y | Y | 7 |
| Tora (2013) | Y | Y | Y | Y | N | Y | Y | Y | 7 |
| Nimani and  Hamdela (2015) | Y | Y | Y | Y | N | N | Y | Y | 6 |
| Manyike et.al (2015) | Y | Y | N | Y | N | N | Y | Y | 5 |
| Seble et.al  (2004) | Y | Y | Y | Y | N | Y | Y | Y | 5 |
| Yaynshet et.al  (2007 | Y | Y | Y | Y | Y | Y | Y | Y | 8 |

**NB: Score =8 high quality (N=4 Studies)**

**Score= 6-7 moderate quality (N=15 Studies)**

**Score < or = 5 low quality (N=5)**
