# Supplementary material for: Gender-based violence among female youths in educational institutions of Sub-Saharan Africa: a systematic review and meta-analysis
Source: Syst Rev. 2019 Feb 25;8:59. doi: 10.1186/s13643-019-0969-9 (PMC6388495; doi:10.1186/s13643-019-0969-9)
Supplement: Supplementary file 3 — Description of studies included in the review. (DOCX 34 kb) [file 13643_2019_969_MOESM3_ESM.docx]

Additional file 3 Description of studies included in the review

|  | Authors and Year | Country | Sample characteristics | Tools | Prevalence and risk factors | Weakness of study |
| --- | --- | --- | --- | --- | --- | --- |
| 1. | Agartha et al. (2012) | Uganda | 633 female university students  (18-24 years) selected using simple random sampling technique among | Self-administered questionnaire | The prevalence of physical, psychological and sexual violence was 7.40%, 26.10% and 31.10% respectively.  Females who perceived exposure to threats of physical violence were significantly associated with experience of sexual coercion [AOR=2.20, 95%CI 1.60-3.0; AOR, 95%CI=1.03-2.80, respectively]. Frequent alcohol consumption was more likely to be associated with sexual coercion, threats of violence and physical violence [AOR=1.80; 95%CI=1.0-3.20; AOR=2.20; 95%CI=1.30-3.70, AOR=1.90, 95%CI=1.10-3.20 respectively]. | - Nothing is known about the frequency/situational aspects of violence, where the violence took place, the identity of perpetrators and severity or threat of violence to which the respondents were exposed. |
| 2 | Ajuwonet al. (2006) | Nigeria | 297 female students (10-24 years) selected by simple random sampling among secondary school students. | Self-administered questionnaires DHS | The prevalence of lifetime sexual coercion was 35%. State [Baunchi AOR=2.26; 95%CI=1.63-5.63, Borno AOR =5.66; 95%CI 3.33-9.90], and having a boyfriend  [AOR=4.19; 95%CI 2.83-6.40] were factors making it more likely that students experienced sexual coercion whereas religion [Islam AOR= 0.35; 95%CI 0.16-6.7], Christianity [AOR= 0.77; 95%CI 0.36-1.45)), living with both parents [AOR= 0.56; 95%CI 0.33-1.03] and living with one parent [AOR= 0.47; 95%CI 0.22-1.27] were factors making it less likely students experienced sexual coercion. | - Relatively small sample size. |
| 3 | Anderson et al. (2012) | 10 southern African countries. | 25,840 females (11-16 years) selected using simple random sampling technique | School Based Health Survey | The prevalence of lifetime experience of sexual violence was 28.80%. Age group 11-13 years [AOR=1.71;95%CI 1.52-1.92], having enough food to last a week in house [AOR=1.51;95%CI 1.35-1.69], a higher proportion of students reporting experiencing sexual violence [AOR=1.84;95%CI 1.61-2.11], a higher proportion of students reporting perpetration of sexual violence [AOR=1.48;95%CI 1.29-1.70] and drinking alcohol [AOR=1.31;95%CI 1.15-1.49] were factors which increased the risk of experiencing lifetime sexual violence. | - The study could not establish causality |
| 4 | Markos et al.  (2014) | Ethiopia | 207 female high school students (18-24 years) selected using simple random sampling | self-administered questions | The prevalence of lifetime sexual violence was 25.0%. Living alone [AOR=4.70; 95%CI 1.0-2.40], living with others [AOR=3.90; 95%CI 1.30-12.0], being in grade 9-10 [AOR=2.70; 95%CI1.20-6.0] and having a boyfriend or girlfriend [AOR=2.70, 95%CI 1.60-4.80] were predictors of sexual violence. | - Relatively small sample size - Social desirability bias and recall bias - Difficult to establish cause and effect relationship |
| 5 | Oheneet al.  (2015) | Ghana | 908 females (12-18 years) selected using simple random sampling | WHO (GSHS) | The prevalence of lifetime sexual and physical violence was 27.50% and 39.10% respectively. Sexual practice with [AOR=2.43;95%CI1.55-3.81] or without [AOR=2.51;95%CI 1.60-3.90] condom use at recent sex, being a victim of bullying [AOR=1.56;95%CI 1.12-2.17], and being cyber bullied [AOR=1.58;95%CI 1.10-2.27] were factors significantly associated with sexual violence victimization whereas friends not being sexually active [AOR=0.53;95%CI 0.34-0.84] and parental monitoring [AOR=0.82;95%CI 0.66-1.00] were protective factors. Alcohol consumption [AOR=2.25; 95%CI 1.2-4.20], attempting suicide [AOR=2.10; 95%CI 1.25-3.54 and bullying others [AOR=1.96; 95%CI 1.55-2.45] were contributing factors for physical violence victimization whereas parent’s respect for privacy [AOR=0.83; 95%CI 0.69-1.00] was a protective factor. | - The findings are not generalized to out- school youths. - The study is based on self-reporting which may lead to under-reporting of sexual violence due to its sensitive nature, a factor which cannot be excluded. - The context and mechanism of violence victimization were beyond the scope of the study. It is difficult to establish a cause and effect relationship due to cross-sectional study design |
| 6 | Wandera et al.  (2017) | Uganda | 1937 female (10-15 years and above) high school students selected using simple random sampling technique | WHO | The lifetime and current prevalence of physical violence was 28.20% and 9.30% respectively. The lifetime and current prevalence of emotional violence was 31.60% and 15.30% respectively. The lifetime and current prevalence of sexual violence was 4.30% and 1.30% respectively. The overall lifetime and current prevalence of GBV was 46.00% and 21.20% respectively. Being older [AOR=0.86;95%CI 0.82-0.91] was a factor less likely to be associated with physical violence, not living with biological parents [AOR=1.17;95%CI 1-10.38], children who shared a sleeping area with one adult [AOR=1.23;1.01=1.50], ever worked for payment [AOR=1.28;95CI 1.07-1.53], having attitude supporting violence from school scores [AOR=1.03;95%CI 1.00-1.05], exposure to interpersonal violence [AOR=1.92;95%CI 1.62-2.27], having a high educational performance [AOR=1.36;95%CI 1.05-1.77] and having a higher SDQ score [AOR=1.05;95%CI 1.03-1.06) were factors associated with lifetime physical violence victimization.  The factors associated with emotional victimization were not living with biological parents [AOR=1.22;95CI1.04-1.41], walking alone [AOR=1.68; 95% 1.06-2.66] or with someone [AOR=1.83;95%CI 1.17-2.86] to school, reporting disability[AOR=1.34;95CI 1.02-1.76], eating one meal only on the previous day [AOR=1.26;95% 1.01-1.57], having ever worked for payment [AOR=1.36;95%CI 1.15-1.62], having a supportive attitude from school staff [AOR=1.04;95%CI 1.02-1.06], exposure to interpersonal violence [AOR=1.29;95%CI 1.09-1.52] and SDQ score [AOR=1.05;95%CI 1.03-1.07] | - The violence measured is perpetrated by peers. Researchers did not assess the power relationship between perpetrators and victims. It is not representative. - Under-reporting and it was unable to establish the relationship between violence and associated factors due to cross-sectional data. |
| 7 | Yabarra, et al.  (2013) | Uganda | 1506 females (12-19+ years) selected using random sampling technique | self-administered questionnaires | The prevalence of sexual violence victimization was 66.0%. Being older [AOR=1.35; 95%CI 1.01-1.82], having a father with primary school education or lower [AOR=3.06; 95%CI 1.12-8.38], social support from family [AOR=0.89; 95%CI 0.80-0.98], very strong chance of getting HIV [AOR=3.46; 95%CI 1.20-10.02] and condom use at last sex [AOR=4.11;95%CI 1.71-9.90] were factors associated with sexual violence victimization. | - It is not known how adolescents in Uganda interpreted the question about coercion. - The issue of representativeness (out of school youths or youths residing in less urban areas did not participate in this study). - Skip patterns confused the students. |
| 8 | Arnold, et al. (2008). | Ethiopia | 1,330 female college students (18-24 years) selected using simple random sampling technique. | WHO Questionnaire for multi-country study for Ethiopia.  Self-administered questionnaires | The overall lifetime and current prevalence of gender-based violence was 59.90% (95%CI 57.20%-62.60) and 40.30% (95% CI 37.50%-43.10%) respectively. Out of the lifetime prevalence GBV, 54.90 % (95% CI 52.0%-57.80%) was sexual violence and 18.5% (95% CI 15.70%-21.60%) was physical violence. Out of the current prevalence of GBV 20.20% (95% CI 16.90%-23.50%) were victims of physical abuse and 57.70% (95% CI 53.30%-62.0%) were victims of sexual abuse. Being of the Protestant religion [AOR=1.80; 95% CI 1.30-2.50), alcohol consumption [AOR=1.70; 95% CI 1.10-2.60], both alcohol and khat consumption [AOR=1.80; 95% CI 1.00- 3.00], and witnessing violence during childhood [AOR=2.20; 95%CI; 6.00-3.10] were factors significantly associated with gender-based violence. Being followers of the Protestant religion [AOR=1.70; 95% CI 1.20-2.40], childhood rural residence [AOR=1.60; 95% CI 1.10-2.30], alcohol consumption [1.80; 95% CI 1.10-2.80], consumption of both khat and alcohol [AOR=2.20; 95% CI 1.30-3.80] and witnessing violence during childhood [AOR=2.40; 95% CI 1.70-3.40] were strongly associated with sexual violence. | - Causality cannot be established due to the cross-sectional study design. Voluntary participation in the study is stressed, which may have implications for the response rate. - The tools were not used previously on college students - Study emphasized individual and relationship risk factors for GBV |
| 9 | Takle Abulie and Tesfaye Setegn (2014) | Ethiopia | 397 female university students  (20-24 years) selected using systematic random sampling technique. | self-administered questionnaires | The prevalence of lifetime and past 12 month sexual coercion was 41.10% and 25.40% respectively. Being aged 17-19 years [AOR=0.24; 95%CI 0.07-0.77] was less likely to be associated with lifetime sexual coercion and consumption of alcohol [AOR=4.20; 1.40-12.66] increased the odds of sexual coercion. | - Not reported |
| 10 | Bekele et al.  (2015) | Ethiopia | 618 female  university students  (18-24 years)  selected using simple random sampling technique | WHO multi-country study questionnaire. Self-administered. | The prevalence of lifetime sexual violence was 10.90%. Having a mother whose educational level was grade 1-4 was less likely to report sexual violence [AOR=0.25;95% CI 0.08-0.76], witnessing parental violence [AOR=5.77;95%CI 2.96-11.23], having a boyfriend [AOR=4.78 ;95%CI 2.0-11.27 and use of khat [AOR=3.11; 95% CI 1.36-7.11] were factors making it more likely students reported sexual violence | - Causality cannot be established due to cross sectional study design. - Responses bias. - Under-reporting of sexual violence. |
| 11 | Bekele et al.  (2014) | Ethiopia | 597 female  university students (20-24 years) selected using simple random sampling technique | WHO multi-country study questionnaire. Self-administered | The prevalence of sexual coercion in lifetime and past 12 months was 76.40% and 43.70% respectively. Childhood residence in rural area [AOR=0.65; 95%CI 0.08-0.89] and having a literate father [AOR=0.17; 95%CI 0.05-0.06] were factors making it less likely that students reported lifetime sexual coercion. Use of alcohol [AOR=1.53; 95%CI 1.62-6.50], parents living together [AOR=6.53; 95%CI 1.38-30.80], witnesses of childhood parental violence [AOR=5.77; 95%CI 2.96-11.23] and multiple sexual partners [AOR=4.32; 95%CI 1.10-16.23] increased the risk of lifetime sexual coercion. | - Causality cannot be established. - This study may be subject to recall bias due to self-reporting. |
| 12. | Benti Tsegaye and Teferi Elias (2015) | Ethiopia | 564 female college students (18-25 years) selected using simple random sampling technique | Self-administered questionnaires | The lifetime prevalence of completed rape, attempted rape and sexual harassment was 20.80%, 23.10% and 41.10% respectively. Having multiple sexual partners [AOR=7.24; 95%CI 3.10-13.12], early sexual debut [9.51; 95%CI 2.29-39.56], receiving little pocket money [AOR=6.95; 95%CI 3.01-16.05, use of alcohol [AOR=2.31; 95%CI 1.13-4.71] and presence of divorced parents [AOR=3.04; 95%CI 1.16-7.95] were predictors of sexual coercion. | This study cannot establish causality due to cross-sectional study design. |
| 13. | Iliyasuet et al.  (2011) | Nigeria | 300 female university students (18-24 years) selected using systematic random sampling technique | Nigeria Demographic and Health survey self-administered questionnaires | The overall prevalence of gender-based violence was 58.80% [95% CI =52.90% -64.50%]. The prevalence of sexual, physical and emotional violence was 22.20%, 22.80%, and 50.8% respectively. Students who were single [AOR=1.51; 95%CI 1.04-3.34], from another ethnic group [AOR=2.10; 95%CI 1.29-5.90], on campus residents [AOR=1.60; 95%CI; 1.28-3.69], Christian [AOR=1.40; 95%CI 1.18-5.99] and part of faculty of social/management sciences [AOR=1.63; 95%CI 1.21-4.96] were more likely to have experienced gender-based violence. | - Study is not homogenous and may not be generalized to general population. - Under-reporting due to fear of perpetrators and age and gender of interviewers. |
| 14 | Mamaru et al.  (2015) | Ethiopia | 385 female university students (18-25 years) selected using systematic sampling technique | self-administered questionnaires | The prevalence of verbal and nonverbal sexual harassment, and physical harassment was 90.4%, 80.0%, and 78.2% respectively. Students who were physically and nonverbally harassed were more likely to experience psychological distress [AOR=3.95; 95CI% 1.98-7.88] and [AOR=12.09; 95%CI 5.19-28.21] respectively. Being part of the College of Public Health and Medicine [AOR=3.07; 95%CI 1.18-8.04] and earning monthly income 100-499 [AOR=10.23; 95%CI 2.89-36.19] were factors associated with physical harassment. | Not reported |
| 15. | Letta et al. (2014) | Ethiopia | 801 female  high school students (15-25 years)  selected using simple random sampling technique | DHS toolkit of USAID 2008-2013 self-administered questionnaires. | The overall prevalence of gender-based violence was 62.20%. The prevalence of sexual, physical and psychological violence was 28.46%, 33.46% and 40.20% respectively. Alcohol consumed by students [A04=3.80; 95%CI 1.43-10.09], living with stepmother/father [AOR=3.79; 95%CI 1.43-10.09], illiterate mothers [AOR=2.13; 95%CI 1.13-4.02 and use of alcohol by parents [AOR=4.73; 95%CI 1.97-11.33] increased the risk of students for physical violence. Students who were divorced [AOR=19.36; 95%CI 6.13-61.13 and married [AOR=8.24; 95%CI 3.48-19.5], alcohol consumption by student [AOR=5.66;95%CI 2.13-9.08], who received financial support from relative [AOR=4.37;95%CI 2.10-9.08], had an illiterate father [AOR=2.22;95%CI 1.28-3.87], alcohol consumption by parents [AOR=3.48;95%CI 1.35-8.96], had mother whose occupation was farmer [AOR=2.72; 95%CI 1.35-5.45] and government employee [AOR=2.38; 95%CI 1.28-4.42] were more likely to have experienced sexual violence. Alcohol used by students [AOR=2.99; 95%CI 1.27-7.00], having a mother whose occupation was farmer [AOR=3.68; 95%CI 1.33-10.17 and private business [AOR=1.76; 95%CI 1.12-2.76] increased the odds of psychological violence. | - Causality cannot be established, - Under-reporting due to sensitive issues, - Recall bias or social desirability bias and - Some students may be dishonest in responding to questions. |
| 16 | Mulluet al.  (2015) | Ethiopia | 140 female high school students (14-24 years) selected using simple random sampling technique | self-administered questionnaires | The overall lifetime and current prevalence of gender-based violence was 67.70% and 57.30% respectively. The lifetime and current prevalence of physical violence was 66.10% and 54.80% respectively. The lifetime prevalence of sexual violence was 24.20%. Having good and above educational achievement [AOR=0.09; 95%CI 0.009-0.88] and not drinking alcohol [AOR=0.38; 95%CI 0.15-0.93] decreased the risk of gender-based violence. Living in a rural residence [AOR=4.85; 95%CI 1.06-22.19] and witnessing violence as a child [AOR=3.70; 95%CI 1.05-12.91] were significantly associated with physical violence. Currently living with boyfriend or married [AOR=4.90; 95%CI 1.02-23.78] and being an alcohol user [AOR=7.30; 95%CI 1.24-23.78] were predictors of sexual violence. | - Relatively small sample size |
| 17. | Mekuria, et al.(2015) | Ethiopia | 369 female high school students (14-19 years) selected using simple random sampling technique | Childhood experience  of care and abuse questionnaire (CECAQ).  Self-administered | The lifetime prevalence of childhood sexual abuse was 11.0%. Never having open discussion about sexuality and reproductive health with parents [AOR=2.93;95%CI 1.33-6.45], having a father of educational status below secondary [AOR=4.69;95%CI 1.84-11.95], living with friends [AOR=3.31;95%CI 1.23-8.89], living alone [AOR=4.30;95%CI 1.81-10.24] and family income <=712.5(37.5 USD) [AOR=3.82;95%CI 1.76-8.31] increased the odds of experiencing lifetime childhood sexual abuse. | - Causality cannot be established. - Under-reporting due to personal issues and sensitive issues related to sexuality. |
| 18. | Shimekaw, et al.(2013) | Ethiopia | 541 female college students (18-24 years) selected using multistage sampling technique | Self-administered questionnaires | The prevalence of sexual violence was 37.30%. The prevalence of sexual harassment and rape was 35.80% and 6.30% respectively. Living in rural residence during childhood [AOR=4.51; 95%CI 1.67-12.16], having peers drinking alcohol [AOR=3.13; 95%CI 1.09-8.97] and students who discussed reproductive health issues with their parents [AOR=4.36; 95%CI 1.40-13.56] were factors significantly associated with sexual violence. | - Not reported |
| 19. | Umana, et al.(2014) | Nigeria | 1355 female  university students (18-24 years)  selected using simple random sampling technique | Self-administered questionnaires | The overall lifetime prevalence of intimate partner violence was 42.30%. The lifetime prevalence of sexual, physical and psychological violence was 6.60%, 7.80% and 41.80% respectively. Being postgraduate [AOR=0.64; 95%CI 0.46-0.87] and married [AOR=0.53; 95%CI 0.35-0.78] reduced the odds of intimate partner violence. Use of alcohol [AOR=2.36; 95%CI 1.82-3.06], cigarette smoking [AOR=2.46; 95%CI 1.58-3.83] and history of interpersonal violence [AOR=2.40; 95%CI 1.88-3.07] were more likely to experience violence. Childhood (before the age 15) experience of sexual, physical, and psychological violence was 9.60%, 28.20% and 6.60% respectively. | - Causality cannot be established. - Recall bias, particularly of experiences during childhood - Reluctant or under-estimated reporting of experience of violence due to social desirability bias |
| 20. | Tora (2013) | Ethiopia | 374 female university students (17-24 years)  selected using simple random sampling technique | self-administered questionnaire | The prevalence of attempted rape, completed rape, physical harassment, verbal harassment, and forced sexual initiation was 23.40%, 8.90%, 24.20%, 18.70% and 11.30% respectively. Students who had experience of sexual intercourse had an increased risk of attempted rape [AOR=0.09; 95%CI 0.04-0.23], completed rape [AOR=0.04; 95%CI 0.01-0.13] and physical harassment [AOR=0.43; 95%CI 0.19-0.94] than those who did not have such experience. | - Study may underreport experiences of victimization due to fear of blame and social stigma. - This study may underestimate, and there may not be enough information to infer sexual victimization of females in other places and cultural settings. |
| 21 | Nimani and  Hamdela (2015) | Ethiopia | 332 female high school students (12-20 years) selected using simple random sampling technique | Self-administered questionnaires | The lifetime and current prevalence of sexual violence was 32.80% and 16.60% respectively. The prevalence of completed and attempted rape in their lifetime was 1.20% and 4.20% respectively. Having a boyfriend/husband [AOR=0.15; 95%CI 0.07-0.30] and experiencing parental violence [AOR=0.49; 95%CI O.29-0.83 were factors making it less likely to have experience of sexual violence. Students who have family supervision [AOR=1.92; 95%CI 1.16-3.17] were significantly associated with sexual violence. | - Not reported |
| 22. | Manyike, et al. (2015) | Nigeria | 267 females (10-24 years) selected using simple random sampling technique | self-administered questionnaires | The lifetime prevalence of child sexual abuse was 34.10%. The predominant form of abuse (18.40%) was watching pornographic films, drawings, videotapes or magazines. Most of the children (11.50%) were abused once at 7-12 years. There was no association between socio-economic class, age and child sexual abuse. | - Study did not address the impact of sexual abuse on adolescents. |
| 23. | Seble, et al.  (2004) | Ethiopia | 612 females (18-24 years) selected using systematic sampling techniques | self-administered questionnaires | The prevalence of lifetime completed, and attempted rape was 12.70% and 27.50% respectively. The prevalence of last year completed and attempted rape was 1.80% and 7.40% respectively. The prevalence of lifetime and previous 12 months any harassment was 58.0% and 41.80% respectively. Having a boyfriend [AOR=2.37; 95%CI 1.43-3.92], being sexually active [AOR=9.95; 95%CI 5.56-17.75] and being divorced or separated or widowed [AOR=2.31; 95%CI 1.10-4.83] were factors making it more likely that students had experience of lifetime rape. Students living with both family [AOR=0.45; 95%CI 0.22-0.92] and students nearly living family as the money sent needed [AOR=0.13; 95%CI 0.10-0.89] were less likely to have experience of lifetime rape. | - Inadequate comparisons due to lack of literature. - The outcome was assessed by the reports of participant. - Under-reporting may occur due to sensitivity of the issues. |
| 24. | Yaynsh,et, et al.(2007) | Ethiopia | 1024 females  (18-24 years) selected using simple random sampling technique | Self-administered questionnaires | The overall prevalence of lifetime and current gender-based violence was 62.10% and 40.20% respectively. The lifetime and current prevalence of sexual violence was 45.40% and 28.10% respectively. The lifetime and current prevalence of physical violence was 46.30% and 26.40% respectively. Having poor academic performance [AOR=2.51; 95%CI 1.29-4.85], living far away from family [AOR=1.53; 95%CI 1.09-2.15], witnessing parental violence as a child [AOR=1.87; 95%CI 1.32-2.66] and having a boyfriend or girlfriend who drinks alcohol [AOR=1.98; 95%CI 1.26-3.11] were factors making it more likely that students experienced physical violence. Having a childhood rural residence [AOR=1.48;95%CI 1.03-2.12], being aged 20-24 years [AOR=1.79;95%CI 1.03-3.312], witnessing parental violence as a child [AOR=1.45;95%CI 1.02-2.06], being sexually active [AOR=1.82;95%CI 1.24-2.67], ever using alcohol [AOR=2.14;95%CI 1.54-2.98] and having female or male friends who drink alcohol increased the likelihood of experiencing sexual violence. Being aged 20-24 years [AOR=1.71;95%CI 1.03-2.85], poor school achievement [AOR=2.09;95%CI 1.10-3.97], having a boyfriend [AOR=1.46;95%CI 1.05-2.02],living far from family [AOR=1.43;95%CI 1.04-1.98],witnessing parental violence as a child [AOR=1.54;95%CI 1.10-2.16], being sexually active [AOR=1.44;95%CI 1.01-2.07],ever drinking alcohol [AOR=1.97;1.43-2.71] and having male or female peers who drink alcohol [AOR=2.63;95%CI 1.65-4.18] increased the risk of experiencing gender based violence. | - The prevalence of gender-based violence may be underestimated due to data collected from survivors or - The study does not include drop outs or absent students. - Causality cannot be established. |
